# High-Temperature Oxidation of Heavy Boron-Doped Diamond Electrodes: Microstructural and Electrochemical Performance Modification

**DOI:** 10.3390/ma13040964

**Published:** 2020-02-21

**Authors:** Jacek Ryl, Mateusz Cieslik, Artur Zielinski, Mateusz Ficek, Bartlomiej Dec, Kazimierz Darowicki, Robert Bogdanowicz

**Affiliations:** 1 Department of Electrochemistry, Corrosion and Materials Engineering, Faculty of Chemistry, Gdansk University of Technology, Narutowicza 11/12, 80-233 Gdansk, Poland; matciesl2@student.pg.edu.pl (M.C.); artzieli@pg.edu.pl (A.Z.); kazdarow@pg.edu.pl (K.D.); 2 Department of Metrology and Optoelectronics, Faculty of Electronics, Telecommunication and Informatics, Gdansk University of Technology, Narutowicza 11/12, 80-233 Gdansk, Poland; matficek@pg.edu.pl (M.F.); bartlomiej.dec@pg.edu.pl (B.D.); robbogda@pg.edu.pl (R.B.)

**Keywords:** boron-doped diamond, high-temperature treatment, surface oxidation, microstructure defects, electrochemical activity

## Abstract

In this work, we reveal in detail the effects of high-temperature treatment in air at 600 °C on the microstructure as well as the physico-chemical and electrochemical properties of boron-doped diamond (BDD) electrodes. The thermal treatment of freshly grown BDD electrodes was applied, resulting in permanent structural modifications of surface depending on the exposure time. High temperature affects material corrosion, inducing crystal defects. The oxidized BDD surfaces were studied by means of cyclic voltammetry (CV) and scanning electrochemical microscopy (SECM), revealing a significant decrease in the electrode activity and local heterogeneity of areas owing to various standard rate constants. This effect was correlated with a resultant increase of surface resistance heterogeneity by scanning spreading resistance microscopy (SSRM). The X-ray photoelectron spectroscopy (XPS) confirmed the rate and heterogeneity of the oxidation process, revealing hydroxyl species to be dominant on the electrode surface. Morphological tests using scanning electron microscopy (SEM) and atomic force microscopy (AFM) revealed that prolonged durations of high-temperature treatment lead not only to surface oxidation but also to irreversible structural defects in the form of etch pits. Our results show that even subsequent electrode rehydrogenation in plasma is not sufficient to reverse this surface oxidation in terms of electrochemical and physico-chemical properties, and the nature of high-temperature corrosion of BDD electrodes should be considered irreversible.

## 1. Introduction

Boron-doped diamond (BDD) surfaces are widely studied due to their unique electrochemical and physico-chemical properties [1]. While the most often reported applications are for sensing [2], energy storage [3], or water treatment [4], BDD does play an important role in high-temperature environments [5]. The electrically conductive nanocrystalline boron-doped diamond (BDD) layers can be applied as a protective coating of Si photoelectrodes in sun-driven photoelectrochemical cells in aqueous electrolyte solutions [6]. Furthermore, wastewater treatment with advanced oxidation processes at BDD usually strongly depends on the potentials and current densities. It has been shown that a larger current promotes stability for urea removal at high potentials [7], which could also induce the thermal issues and electrode surface defects [8]. Moreover, the relatively high thermal conductivity coefficient (~700 W/mK) of BDD allows for its use as a heat spreader, replacing the commonly used metal spreaders such as copper, copper/refractory, or copper laminate in high power RF/microwave devices resulting in higher isolation of the ground plane at below 1.5 GHz [9,10].

BDD thin films are found to be particularly attractive as electrodes for electrolysis and electroanalytical applications due to their outstanding properties, which are significantly different from those of other conventional electrodes (e.g., glassy carbon or platinum electrodes [11]). In addition to the innate properties of diamond, such as high thermal conductivity, high hardness, and chemical inertness, the attractive features of conductive BDD include a wide electrochemical potential window in aqueous and non-aqueous media, very low capacitance, and extreme electrochemical stability [12]. BDDs are said to possess higher oxidation resistance at elevated temperatures in comparison to pure diamond [13], which is due to the formation of a B_2_O_3_ surface layer. Wang and Swain [14] reported a Pt/diamond composite electrode that exhibited superb morphological and microstructural stability during vigorous electrolysis in acidic media at a temperature of around 170 °C and current density around 0.1 A/cm^2^. In addition, BDD layers can operate at high temperatures in electronics, an example being diamond-based Schottky barrier diodes for high-power devices [15,16].

High-temperature treatment is also one of the reported routes to modify and oxidize the surface termination of BDD electrodes [17,18,19]. Other approaches include oxidation under electrochemical polarization [20,21], chemical agents [22,23], ozone [24], plasma and/or UV treatment [25,26], but also natural aging in air [27,28,29]. The surface termination type significantly differentiates the electric and physico-chemical properties of boron-doped diamond electrodes. Hydrogen termination (HT-BDD) leads to hydrophobic and non-polar behavior, as well as high surface electrical conductivity and low electric transfer resistance [22,25]. The presence of hydroxyl or carbonyl surface bonds and oxidized BDD termination (OT-BDD) results in hydrophilicity and much lower surface electrical conductivity [30,31]. On the other hand, the electrochemical potential window for OT-BDD electrodes is reported to be wider than in the case of hydrogen-terminated ones [31,32]. Importantly, Zielinski et al. [29] reported that oxidation homogeneity of polycrystalline electrodes as well as oxidation rate in general highly depend on the above-mentioned modification route and confirmed that various treatment procedures produce different surface terminating species (C–OH, C–O-C, C=O, C–OOH, etc.), which may have a significant influence on tailoring desired functionalization procedures and BDD electrode characteristics.

Among the multitude of reported oxidation routes, high-temperature oxidation is characterized by a few unique features [29]. First of all, it appears to be the most heterogeneous at low durations, which was claimed to be related to the diverged propensity of differently oriented BDD crystal planes towards surface oxidation. Furthermore, according to studies where X-ray photoelectron spectroscopy (XPS) was performed, high-temperature oxidation tends to produce a significantly larger amount of surface hydroxyl species than any other reported approach. Their high polarity in comparison to other surface species results in very small reported contact angles during drop shape analysis tests. Next to electrochemical anodization, high-temperature treatment is one of the most often used pretreatment methods to clean the impurities on as-prepared BDD electrodes after the chemical vapor deposition (CVD) process [19,33].

High-temperature oxidation may also lead to changes in surface morphology as well as the altered sp^2^/sp^3^-carbon ratio [34,35]. The surface structure of BDD electrodes exposed to elevated temperatures in an oxygen-containing atmosphere was presented by Jiang et al. [17], who noticed that during such a treatment the amorphous carbon phase converts to the diamond phase and that the diamond grain increases with the decrease of grain boundaries. In addition, the short high-temperature treatments (30 min) led to the arrangement of grain boundaries on the electrode surface, which had a positive effect on their conductivity. An interesting consequence of the high-temperature oxidation is the enhancement of the peak ratio between the diamond peak and the graphitic peak, showing a decreasing amount of sp^2^-carbon after the process, resulting in an increased carrier concentration [36,37]. However, prolonged high-temperature oxidation led to disordered grain boundaries and significantly deteriorated conductivity of the BDD electrode. High-temperature treatment may also lead to a diffusion of silicon substrate through the columnar structure of the diamond film and corrosion [33].

In light of the above presented discussion, the aim of this work was to present the effect of high-temperature treatment in air at 600 °C on the utility properties of boron-doped diamond electrodes, in particular for electrochemical applications. To the best of our knowledge, there are no dedicated studies on BDD electroactivity and stability of the properties when subjected to prolonged high-temperature exposure, the homogeneity and the rate of high-temperature corrosion of BDD, and its reversibility.

## 2. Materials and Methods

BDD films were synthesized in a microwave plasma-assisted chemical vapor deposition system (SEKI Technotron AX5400S, Tokyo, Japan). The substrates were seeded by sonication in nanodiamond suspension for 30 min following the standard procedure [38]. The chamber stage was maintained at 700 °C during the deposition process and the growth time was 6 h. The boron level expressed as [B]/[C] ratio in the gas phase was 10,000 ppm (boron dopant concentrations 2 × 10^21^ atoms cm^−3^) [39]. A more detailed description of the thin film synthesis can be found elsewhere [40,41]. Then, the electrode surface was cleaned and hydrogenated. First, metallic impurities were dissolved in hot aqua regia (HNO_3_:HCl/1:3), followed by the removal of organic impurities by hot “piranha” solution (H_2_O_2_:H_2_SO_4_/1:3) at 90 °C. Microwave hydrogen plasma treatment was performed using 1000 W microwave power and 300 sccm of hydrogen gas flow for 10 min.

The high-temperature treatment was carried out in an MRT-20 furnace (Czylok, Jastrzebie-Zdroj, Poland). The BDD electrodes were treated at 600 °C for 3, 10, 30, or 90 min in air. Afterward, the samples were removed from the furnace and cooled in air. A similar procedure was carried out in other studies [29,30,33]. After the electrochemical and physico-chemical examination, the hydrogenation procedure described earlier was carried out for high-temperature-treated BDD samples in order to determine surface oxidation reversibility.

Electrochemical measurements were carried out in a three-electrode cell. The working electrode was Si/BDD, with Ag/AgCl used as a reference electrode and platinum mesh as a counter electrode. All reagents were analytical purity (Sigma-Aldrich, Saint Louis, MO, USA). The exposed BDD sample area was 0.25 cm^2^. Cyclic voltammetry (CV) studies were carried out in the polarization range between −0.9 V and 1.1 V vs. Ag/AgCl, at different scan rates between 5 and 800 mV/s. The electrolyte used was 0.5M Na_2_SO_4_ with 2.5 mM K_3_[Fe(CN)_6_] and 2.5 mM K_4_[Fe(CN)_6_]. Scanning electrochemical microscopy (SECM) studies were performed using a three-step motor system (Sensolytics, Bochum, Germany) with 1 μm resolution in each direction, coupled to an Autolab 302 N potentiostat equipped with bipotentiostat module (Metrohm, Herisau, Switzerland). The commercially available ultramicroelectrodes (UMEs) made of platinum wire sealed in glass were used, with a platinum disc diameter of 5 μm. Each probe was polished and rinsed with acetone prior to the study. The electrolytic solution was composed of 2.5 mM K_4_[Fe(CN)_6_] and 0.5M Na_2_SO_4_, purged with argon prior to BDD sample examination. The potential applied to the UMEs and the BDD sample was +0.4 and 0.0 vs. Ag/AgCl, respectively. In such a setup, the oxidation of redox species occurs at the UME tip. The SECM maps were recorded with a scanning speed of 5 μm/s and 1 μm steps in the x-y plane. A similar experimental approach was previously applied by authors [40].

Topographic and electrical microscopic measurements were made using an NTegra Prima device by NT-MDT (Moscow, Russia) in contact mode. CTD-NCHR-10 probes from Nanosensors (Neuchatel, Switzerland) were used with the following catalog parameters: L × W × T lever dimensions: 125 × 29 × 4 µm. Lever spring constant was equal to 71 N/m. The contact force determined from the approach curve was 8 µN. The tip radius of the curvature was in the 100–200 nm range according to manufacturer data. Conductivity measurements were made in the scanning spreading resistance mode using a constant voltage of 20 mV. Scanning electron microscopy (SEM) micrographs were taken with an S-3400 N microscope (Hitachi, Tokyo, Japan), with 20 kV accelerating voltage and operating in secondary electron mode.

High-resolution X-ray photoelectron spectroscopy (XPS) analyses were carried out in C1s binding energy range using an Escalab 250Xi multispectroscope (ThermoFisher Scientific, Waltham, MA, USA). The spectroscope was equipped with a monochromatic Al Kα energy source. The applied X-ray spot diameter was 650 μm and the pass energy through the hemisphere analyzer was 10 eV. Prior to operation, the spectroscope was calibrated on Cu and Au single crystals. Charge compensation was controlled through low-energy electron and Ar+ ion flow by means of a flood gun. Spectral deconvolution was performed with Avantage software (v5.973, ThermoFisher Scientific, Waltham, MA, USA) provided by the spectroscope manufacturer.

## 3. Results and Discussion

The heavy boron-doped diamond electrodes were subjected to electrochemical and physico-chemical examination in order to evaluate the effect of oxidation under high temperature on the charge transfer kinetics. Afterward, the oxidation and corrosion reversibility was evaluated and determined.

### 3.1. Electron Transfer through High-Temperature-Treated BDD Interface

**Figure 1:**
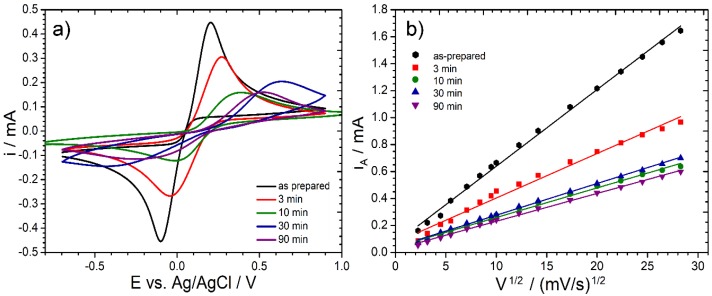
Cyclic voltammetry (CV) studies for boron-doped diamond (BDD) electrodes after high-temperature oxidation at 600 °C. (**a**) CV scans recorded at 50 mV/s for different oxidation durations; (**b**) CV anodic peak current vs. scan rate square root function for each studied electrode. Electrolytic solution: 0.5 M Na_2_SO_4_ with 2.5 mM K_3_[Fe(CN)_6_] and 2.5 mM K_4_[Fe(CN)_6_].

Figure 1 shows the results of the cyclic voltammetry studies, which were carried out on BDD electrodes before and after high-temperature oxidation in air at 600 °C. The redox couple used within this study, [Fe(CN6)]^3−/4−^, is characterized by an inner-sphere electron transfer (ISET) mechanism, which is said to be more dependent on the electrode homogeneity and applied modification procedures, thus offering a more sensitive approach to track subtle changes of charge transfer kinetics [42]. Figure 1a reveals [Fe(CN6)]^3−/4−^ oxidation/reduction kinetics for each sample, observed at a 50 mV/s scan rate. High-temperature oxidation leads to hindered charge transfer kinetics, demonstrated by the decrease in peak current i_A_ and i_C_ when comparing to the as-prepared BDDs, a feature observed even at the shortest treatment duration. The peak current decreased nearly by a factor of 2 after merely 3 min of sample exposure to high temperature, and by a factor of 4 after 10 min. Detailed analysis is presented in Table 1.

It should also be noted that the modification of BDD surface termination type translates into a significant peak separation ∆E increase, the parameter which is inseparably connected with reversibility of the corrosion process. In the case of a single electron transfer reaction, the fully reversible processes should have ∆E equal to 59 mV [43]. The as-prepared BDD sample is characterized by ∆E value of approximately 350 mV. The value is quite high taking into consideration other reported heavy boron-doped electrodes, which is due to the lack of any other electrode pretreatment procedures [41,44]. Nevertheless, the ∆E increase resulting from the applied high-temperature treatment should be explained by the move further away from the diffusion-controlled mechanism, due to the slowing down of the charge transfer kinetics. Prolonged high-temperature treatment is also characterized by the increased anodic and cathodic peak asymmetry, indicating a change in process kinetics, a feature typical for irreversible processes.

The discussed irreversibility of the studied redox process, as well as the decrease in electrode kinetics, are a testimony for surface modification from HT- to OT-BDD as a result of high-temperature treatment. The above-mentioned behavior results in BDD electrode corrosion, causing variability in electrode behavior and worsening its efficiency in electroanalytical studies.

The CV anodic peak vs. the scan rate square root function in the wide scan rate range is illustrated in Figure 1b. These plots show a strong linear trend, with local deviations explained by the heterogeneous nature of the polycrystalline electrodes [45]. To determine the standard reaction rate constant k^0^, a numerically determined current function is used, depending on peak separation. The standard rate constant was calculated with the approach proposed by Velasco [46] to estimate completely irreversible processes. The following equation was used:

(1) $$k^{0}=2.415\exp\left(-0.02\frac{F}{RT}\right)D^{\frac{1}{2}}{\left(E_{p}-E_{\frac{p}{2}}\right)}^{-\frac{1}{2}}v^{\frac{1}{2}}$$

where *E_p_* and *E_p_*_/2_ are the potentials of the CV peak and half-peak, respectively, and v refers to the rate of change of potential; the diffusion coefficient was assumed as D = 6.67 × 10^−6^ cm^2^/s [47].

**Figure 2:**
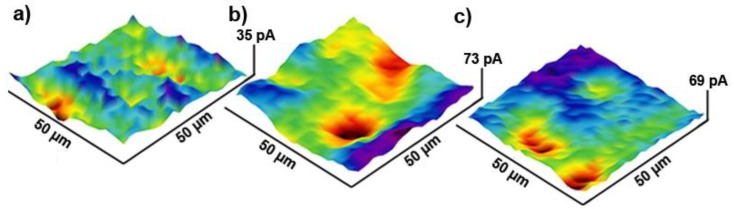
Typical scanning electrochemical microscopy (SECM) maps revealing charge transfer heterogeneity at heavy-doped BDD electrode interface after (**a**) 10 min, (**b**) 30 min, and (**c**) 90 min of high-temperature oxidation at 600 °C in air. Electrolyte: 0.5 M Na_2_SO_4_ + 2.5 mM K_4_[Fe(CN)_6_].

Figure 2 reveals the effect of high-temperature treatment on the local distribution of charge transfer kinetics during [Fe(CN)_6_]^4−^ oxidation, assessed with scanning electrochemical microscopy (SECM). The relative current values strongly depend on the distance between the SECM tip and the electrode and vary from sample to sample. The current value is less important than its local changes due to electrode charge transfer heterogeneity. A normalization procedure was applied for the Z-axis of these graphs in order to show local discrepancies in the oxidation currents. The procedure based on a negative shift of the tip current displayed on each map to the position where the lowest recorded value equaled zero. A similar procedure was successfully applied in previous studies on heterogeneous charge transfer through the BDD electrode [40].

The obtained SECM micrographs clearly illustrate that the surface distribution of the oxidation currents is significantly increasing already after a short 10 min high-temperature treatment (Figure 2b) as compared to the as-prepared BDD electrode (Figure 2a). The heterogeneous electron transfer results from an altered propensity towards surface oxidation by BDD grain of various crystallographic orientations. A similar observation was previously reported for various methods of BDD surface treatment, in particular through electrochemical anodic polarization [40]. Importantly, increasing the high-temperature treatment length does not significantly increase surface homogeneity. Large tip current discrepancies are still observed after 90 min of oxidation (Figure 2c). At the same time, the as-prepared BDD electrode reveals positive feedback on the approach curve, suggesting low charge transfer resistance at the electrode interface [48]. The longer the high-temperature treatment, the higher was the negative feedback observed, whereas the sample oxidized for 10 min was characterized with large discrepancies of the approach curve shapes, depending on tip landing location. These results corroborate the decrease of the electron transfer kinetics with high-temperature treatment duration and the heterogeneous nature of the oxidation process.

### 3.2. High-Temperature Oxidation Influence on BDD Physico-Chemical Properties

**Figure 3:**
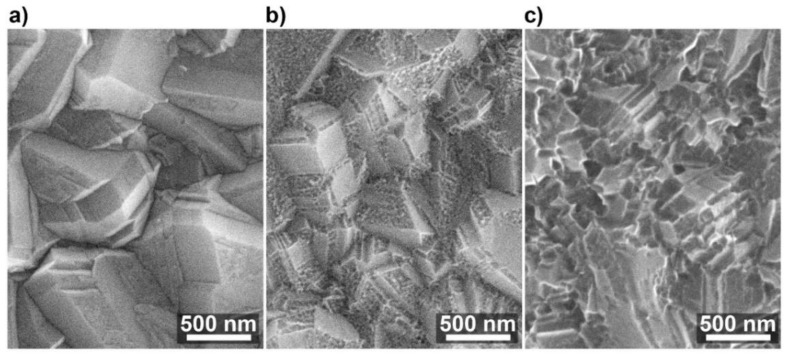
Typical scanning electron microscopy (SEM) micrographs of heavy-doped BDD surface after (**a**) 10 min, (**b**) 30 min, and (**c**) 90 min of high-temperature oxidation at 600 °C in air.

The effect of high-temperature oxidation and corrosion on the microstructure of boron-doped diamond electrodes is shown on the SEM micrographs in Figure 3. The first 10 min of exposure to 600 °C do not lead to significant changes in grain structure; however, after this period the material undergoes gradual degradation.

After 30 min of high-temperature oxidation, small and shallow etch pits start to appear throughout the diamond surface (Figure 3b), yet the grain structure is still recognizable. It is said that diamond can react with O_2_ and water vapor contained within the atmosphere, to create surface etch pits [37]. Treatment in the oxygen-containing environment leads to high BDD surface etching and corrosion in the relatively short periods and a large surface area with respect to the geometric electrode area [49].

Furthermore, it is evident that certain crystallographic planes on the diamond surface are more prone to surface etching. Ohashi et al. [50] reported steam-activated high-temperature treatment in 600–900 °C to be an efficient nanotexturing tool for diamond surfaces. Notably, it was observed that, in the case of heavy-doped BDD films, the (111) facets are more prone to etching [19]. This observation lies in compliance with the fact that (111) facets have the dominant presence in the texture of studied BDD electrodes [51,52]. Other studies revealed that the propensity towards the modification of BDD surface termination is also dependent on crystallographic orientation [40,51].

Extended exposure at elevated temperature leads to advanced electrode decay, where the grain structure of the polycrystalline electrode almost completely fades away after 90 min of high-temperature oxidation (Figure 3c). Overall, the combined effect of surface area enhancement as well as previously reported [36,37] increased charge carrier concentration within the diamond structure may possibly result in an improved electrochemical response by BDD electrodes.

**Figure 4:**
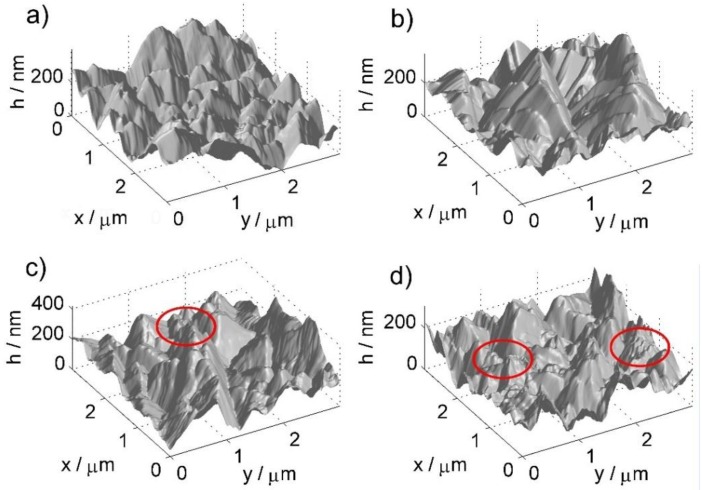
The atomic force microscopy (AFM) topography maps for exemplary BDD samples subjected to high-temperature treatment for different durations: (**a**) reference, untreated sample; (**b**) 10 min; (**c**) 30 min; (**d**) 60 min.

**Figure 5:**
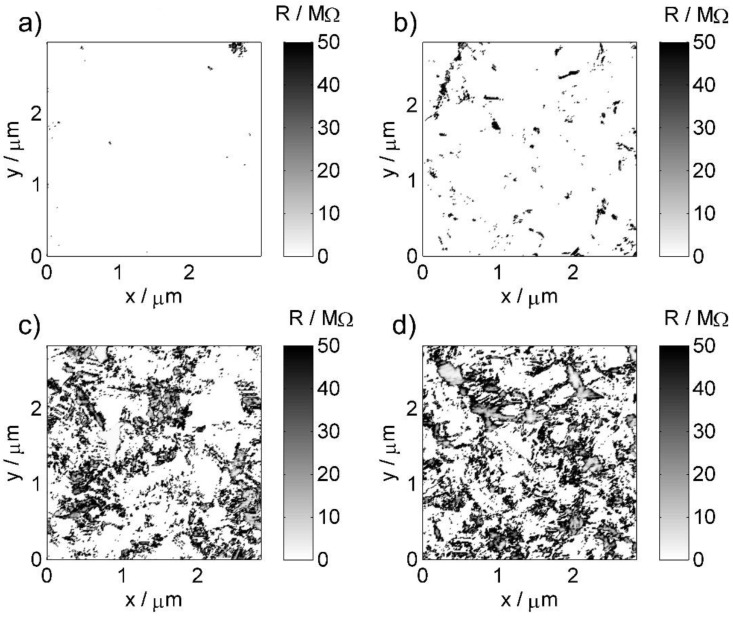
Typical spreading resistance (SSRM) maps for BDD samples subjected to different durations of high-temperature treatment in air at 600 °C: (**a**) reference untreated sample; (**b**) 10 min; (**c**) 30 min; (**d**) 90 min.

The detailed topography studies were carried out using atomic force microscopy (AFM) in contact mode. Next, scanning spreading resistance microscopy (SSRM), an AFM technique derivative, allows obtaining maps of local distribution of surface resistance, originating from changes in BDD termination type due to high-temperature treatment. The topographic images (Figure 4) were acquired simultaneously with surface conductivity maps (Figure 5).

The sequence of three-dimensional images of the surface shown in Figure 4 allows observing the evolution of surface morphology consistent with that obtained by scanning electron microscopy (refer to Figure 3). The first clearly represented pyramidal crystallites change into fractured, irregularly shaped structures with high-temperature treatment duration, clearly visible in the red-marked areas. It should be noted that this form of surface development affects the modification of the conductivity distribution through the geometric factor of the contact surface between the surface and various parts of the surface of the AFM pyramid tip [29]. Furthermore, possible surface development increases might contribute to the increase of the electrochemically active surface area.

For the statistical description of the sample topography, the average roughness parameter was used, defined as:

(2) $$S_{a}=\frac{1}{MN}\overset{M-1}{\underset{k=0}{\sum }}\overset{N-1}{\underset{l=0}{\sum }}\left|z\left(x_{k},y_{l}\right)-\mu \right|$$

where *M* = *N* = 256 are the length and width of the analyzed image in pixels and *µ* is the average height, defined as:

(3) $$\mu =\frac{1}{MN}\overset{M-1}{\underset{k=0}{\sum }}\overset{N-1}{\underset{l=0}{\sum }}z\left(x_{k},y_{l}\right)$$

The above statistical parameters were determined for areas of 3 × 3 µm, the scan was repeated 4 times for each sample in different places, and the average roughness contained in Table 2 is the average value from each set of topographic scans.

The value defined in Equation (2) as *S_a_* determines the local height deviations from the average value for a given area. Maxima can be associated with the presence of high crystallites; however, their degradation, which progresses over the course of the heat treatment process, causes their degradation and additionally the formation of fragments of relatively small sizes, contributing to the reduction of maxima.

Figure 5 reveals the results of surface conductance analysis in accordance with the previously presented assumptions. The SSRM technique makes it possible to create a map of the quantity defined as the spreading resistance [53], characterizing the local changes in nanocontact conductivity between the probe tip and the sample surface. A simplified, commonly used formula [54]:

(4) $$R_{s}=\;\frac{1}{4\sigma a}$$

where *a* is the radius of curvature of the probe tip and *σ* is the specific conductivity of the sample material, indicates the need for a trade-off between spatial resolution and probe durability, related to the thickness of the conductive layer and the radius of curvature a. The probes used in this report have a significantly increased radius of curvature for typical topographic ones (10 nm); however, they provide sufficient resolution to visualize changes in conductivity structure.

There is a visible decrease in the surface resistance average value, as well as a significant change in the mutual relationship between areas with relatively high and low electrical conductivity. For samples subjected to longer exposure, a bimodal character of conductivity can be seen with a gradual increase in the share of low conductivity areas. The observable changes present a good representation of the transition from HT- to OT-BDD under the oxidation agent [29]. Furthermore, it may be observed that high-temperature treatment leads to significant heterogeneity in surface oxidation, where the areas of altered spreading resistance are corresponding to different grain areas of the polycrystalline electrode. This observation may suggest that a varied crystallographic orientation is affecting the oxidation propensity, thus translating to locally variable electrochemical activity, as demonstrated by SECM studies.

It should be noted that the recorded heterogeneity in conductive regions can only be treated as a rough estimate of the variable electrochemical activity due to the difference in the conditions on the nanocontact in the atmosphere of air and in an electrolytic environment. Nevertheless, changes in the spreading resistance should to some extent correlate with changes in the charge transfer resistance on the surface of BDD and additionally reflect the trend of its changes together with the variability of heat treatment conditions. In this sense, the above discussed heterogeneity may correspond to the Compton model of partially blocked electrodes [55,56].

**Figure 6:**
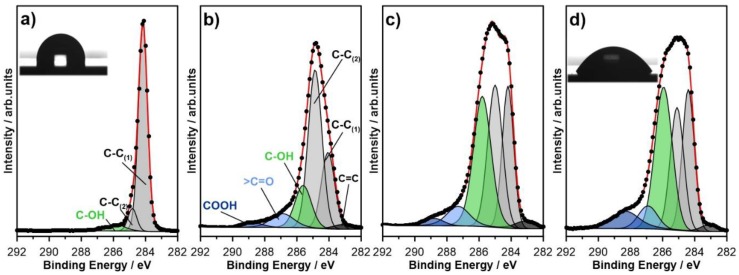
High-resolution X-ray photoelectron spectroscopy (XPS) spectra recorded in C1s binding energy range with applied spectral deconvolution. Spectra recorded for BDD samples: (**a**) as-prepared and after high-temperature treatment in air at 600 °C: (**b**) 3 min; (**c**) 10 min; (**d**) 30 min.

The high-resolution XPS spectra of BDD electrodes after different durations of high-temperature treatment are presented in Figure 6. These data were recorded in C1s peak binding energy range. The spectra were then deconvoluted according to the fitting model presented below and the results of the analysis summarized in Table 3.

The C1s spectra recorded for the as-prepared BDD electrode, not subjected to high-temperature oxidation, reveals relatively simple surface chemistry. The registered spectra may be deconvoluted using three separate components. The primary peak, denoted as C-C_(1)_ and located at approximately 284.2 eV, lies in the energy range characteristic for sp^3^-carbon CH species on the hydrogen-terminated diamond surface and sp^3^-carbon within the BDD bulk. The exact location of this peak depends on crystallographic texture rather than boron dopant concentration [57,58]. Thus, the peak position is in good agreement with previous studies on heavy boron-doped diamond substrates [20,29,59]. The second notable component, C-C_(2)_, is usually attributed to non-hydrogenated carbon atoms on the BDD surface but also adsorbed polyhydride carbon (CH_x_) species [20,60]. The position of this component is typically shifted by +0.7 eV vs. C-C_(1)_, which is also in this case.

The oxidation of BDD surface termination, occurring as a result of high-temperature treatment in air, results in the substitution of hydrogenated terminal bonds with oxygen-containing species: hydroxyl C–OH (peak at 285.6 eV), but also carbonyl >C=O (at 287.0 eV) and carboxyl COOH (at 288.7 eV) groups [41,57,61]. The XPS analyses confirmed that hydroxyl species are the dominant ones on the surface of high-temperature-treated BDD electrode, unlike other types of surface oxidation treatments (electrochemical, oxygen plasma, ozone treatment, etc.) [29]. The HT- to OT-BDD transition is bound to the increase in surface hydrophobicity, as illustrated by the drop shape analyses in the inset of Figure 6a,d.

The total share of oxidized BDD surface area, OT-BDD, counted as a sum of the above-mentioned components is naturally increasing with the duration of sample exposure to the high temperature. The share of OT-BDD surface for the untreated samples was 5.7% and gradually increased up to 43.1% after 10 min of exposure. Prolonged treatment leads to further surface oxidation, but the total OT-BDD share tends to the plateau with only 52.4% oxidized surface after 90 min of treatment. This effect is partially bound to the fact that the XPS analysis is partially obtained from ~5 nm volume beneath the electrode surface. However, similar studies on BDD electrodes, but oxidized by electrochemical or oxygen plasma treatment, have led to significant diminishing of C-C_(1)_ peak, down to 12%–14% [29]. Retaining around 25% of HT-BDD surface even after 90 min of high temperature is well represented and imaged on the SSRM micrographs (see Figure 5), which reveals a significant heterogeneity of oxidized areas on the BDD electrode surface. Following the electric BDD spatial heterogeneity is the electrochemical behavior, determining the conditions of the charge transfer mechanism by a partially blocked electrode.

It was also observed that prolonged high-temperature treatment durations have led to the disappearance of the spectral component, located at approximately 283.2 eV, and originating from surface sp^2^-carbon, which is well explained by a previously mentioned theory that such an exposure results in disordered grain boundaries and significantly deteriorated BDD conductivity.

**Figure 7:**
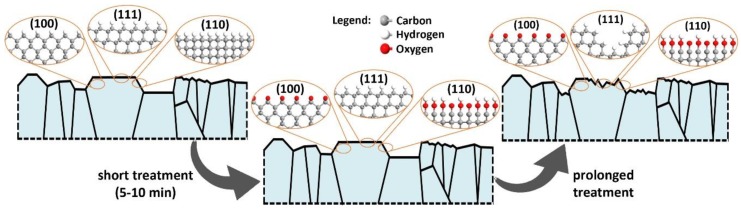
Schematic visualization of the oxidation process under high-temperature treatment in air at 600 °C.

The above discussed effect of high-temperature treatment in air at 600 °C on BDD surface chemistry is schematically presented in Figure 7. The initially hydrogen-terminated surface of the polycrystalline BDD electrode undergoes surface oxidation over very short durations, even after a few minutes of exposure to the oxidation agent. This effect is heterogeneous in nature and hypothetically limited to specific crystallographic planes, based on previous studies [29,51,62]. The authors did not investigate the exact planes of high-temperature interaction within this study. The prolonged treatment leads to the appearance of etch pits on the diamond surface. This effect is said to increase the electroactive surface area; however, it was not confirmed since the OT-BDD surface is characterized by lower values of the standard reaction rate constant. Most importantly, the appearance of the etch pits is heterogeneous and depends on the crystallographic orientation, which is similar to surface oxygen termination. According to the literature survey, the etch pits are primarily initiated at (111) facets [19], which, on the other hand, are the least prone to surface oxidation [63].

### 3.3. Reversibility of the High-Temperature BDD Surface Oxidation

A few available routes for surface rehydrogenation have been reported in the literature, where hydrogen plasma treatment is claimed to be the most efficient [64]. Despite growing awareness and interest in this topic, some studies reveal issues with the reversibility of oxidized BDD electrodes [31]. Various surface physico-chemical properties (such as surface chemistry or contact angle) are often reported to be on par with those observed for BDD electrodes prior to their oxidation; however, electrochemical and electric parameters seem to be a subject of irreversible change. The above-mentioned characterization is most often discussed in the case of electrochemically oxidized BDDs. We decided to evaluate the reversibility of the oxidation process under study since both the mechanism of oxidation and the resultant surface chemistry are claimed to be different in the case of high-temperature treatment than any other oxidation route.

**Figure 8:**
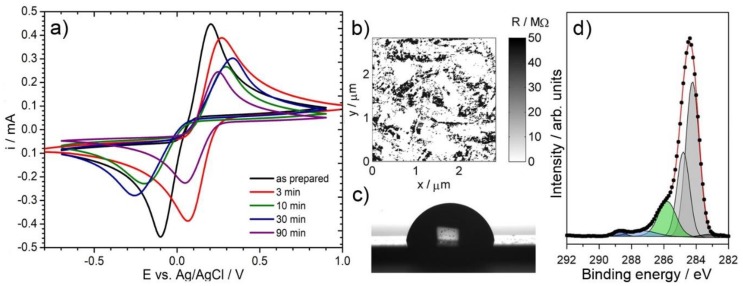
The effect of BDD surface rehydrogenation in plasma: (**a**) CV studies after various durations of oxidation. A scan rate of 50 mV/s. Electrolytic solution: 0.5 M Na_2_SO_4_ with 2.5 mM K_3_[Fe(CN)_6_] and 2.5 mM K_4_[Fe(CN)_6_]; (**b**–**d**) exemplary results for electrodes after 30 min oxidation followed by rehydrogenation: (**b**) SSRM map, (**c**) contact angle analysis, and (**d**) high-resolution XPS C1s spectrum.

Figure 8a reveals the CV scans at 50 mV/s for high-temperature oxidized (at various durations) and then plasma rehydrogenated BDDs, compared to the as-prepared BDD electrode. Our studies show that neither of the investigated electrodes is characterized by improved peak current values. On the other hand, a significant improvement is visible when compared to oxidized BDD electrode kinetics. It appears that very short high-temperature treatments might lead to a certain improvement in electrochemical characteristics, that is, the peak separation ∆E is significantly improved and closer to the theoretical value of diffusion-controlled electrode processes, with high peak symmetry and peak currents equal to nearly 90% of its original value. This feature might be explained by the removal of sp_2_-carbon species adsorbed on the BDD electrode surface after the CVD process, demonstrating a successful electrode cleaning. The longer the high-temperature treatment duration, the lower the value of both anodic and cathodic peak currents and the more irreversible the character of the oxidation process. This effect is naturally connected with the degradation of the BDD grain structure. While the deterioration of the electrode kinetics is progressive, an interesting feature may be observed for the sample after 90 min oxidation and rehydrogenation, which is characterized by the most narrowly observed ∆E value. Following the previously defined partially blocked electrode mechanism, it may be concluded that deep etching of BDD grains (refer to Figure 3c) leads to homogenization of the electrode surface and unification of the diffusion fields at the electrode interface, a conclusion supported by SSRM studies. The detailed analysis is summarized in Table 4.

A similar observation is revealed by SSRM maps, shown in Figure 8b for BDD electrodes after 30 min of high-temperature treatment and rehydrogenation. While the hydrogen plasma is increasing the surface conductance, the Rs is far off from its original value (10.416 MΩ) and the local distribution of electric properties on the BDD surface remains heterogeneous. This is an important observation, confirming that not only the electrochemical but also the electric parameter characteristics have been modified. However, the rehydrogenation process undoubtedly does have a positive effect on BDD surface chemistry, demonstrated by the XPS analysis (Figure 8d). The hydrogenation process led to a significant reduction in the OT-BDD share (from 49.9% to 20.6%) and, in particular, in the number of surface hydroxyl groups (from 35.6% to 15.7%), which decreased more than twice for prolonged oxidized samples. The reappearance of the hydrogen-terminated surface resulted in the increase of surface hydrophobicity, observed with the drop shape analysis (Figure 8c). However, the process is not fully reversible, thus leading to a conclusion regarding the corrosive nature of the high-temperature treatment, in particular at prolonged oxidation durations.

## 4. Conclusions

In summary, we performed a detailed study of the influence of high temperature on the oxidation processes for heavy boron-doped diamond electrodes. Electrode surface was investigated by both electrochemical and physico-chemical techniques to reveal its charge transfer kinetics and oxidation behavior. 

High-temperature oxidation leads to:

✓ hindered charge transfer kinetics, demonstrated by the decrease in peak current on CV scans when comparing to the as-prepared BDDs, a feature observed even at the shortest treatment duration;
✓ the modification of BDD microstructure and the appearance of small and shallow etch pits throughout the diamond surface, damaging diamond structure at prolonged treatments;
✓ the disappearance of the spectral component, located at approximately 283.2 eV, and originating from surface sp^2^-carbon in XPS;
✓ the bimodal character of BDD conductivity observed by SRRM with a gradual increase in the share of low conductivity areas, dependent on the BDD crystallographic structure.

The change in the electrode’s surface electric properties by local surface oxidation is reflected in a more heterogeneous distribution of the diffusion field, observed by SECM. The longer the high-temperature exposure duration, the lower the value of both anodic and cathodic peak currents and the more irreversible is the character of the oxidation process, which is mainly attributed to the corrosion of BDD grain structure and the deterioration of electrochemical activity.

## Figures and Tables

**Table 1:** Electrochemical properties (anodic peak current i_A_, anodic-to-cathodic peak current ratio i_A_/i_C_, peak separation ∆E) based on CV studies (at 50 mV/s scan rate) of BDD samples after various high-temperature treatment durations.

Parameter	Untreated	High-Temperature Treatment			
		3 min	10 min	30 min	90 min
i_A_/mA	0.44	0.29	0.16	0.20	0.16
i_A_/i_C_/-	0.97	1.14	1.33	1.45	1.43
ΔE/V	0.35	0.31	0.39	1.04	0.72
k_0_/cm/s	3.58 × 10^−3^	1.96 × 10^−3^	1.94 × 10^−3^	1.78 × 10^−3^	1.91 × 10^−3^

**Table 2:** Statistical parameters: average roughness S_a_ and mean surface resistance R of studied BDD samples, based on AFM and scanning spreading resistance microscopy (SSRM) analyses.

Parameter	Untreated	High-Temperature Treatment			
		3 min	10 min	30 min	90 min
Mean Sa/nm	55.1374	55.860	51.4811	54.4982	36.3451
Mean Rs/Ω	0.1505	0.7993	1.6417	13.0889	16.1148

**Table 3:** Chemical composition (in %) of various carbon chemical states on the surface of untreated BDD electrodes and after high-temperature treatment in air at 600 °C, based on high-resolution XPS analysis.

Chemical State		BE/eV	Untreated	High-Temperature Treatment			
				3 min	10 min	30 min	90 min
**C1s**	**C-C sp^2^**	**283.2**	2.1	2.4	2.9	1.8	1.5
	C-C_(1)_	284.2	83.5	23.1	23.7	24.4	25.3
	C-C_(2)_	284.9	8.7	49.2	30.3	23.9	20.8
	C-OH	285.6	4.8	15.8	31.6	35.6	36.8
	>C=O	287.0	0.6	7.4	6.6	6.4	6.5
	COOH	288.7	0.3	2.1	4.9	7.9	9.1

**Table 4:** Electrochemical properties (anodic peak current i_A_, anodic-to-cathodic peak current ratio i_A_/i_C_, peak separation ∆E) based on CV studies (at 50 mV/s scan rate) of BDD samples after various high-temperature treatment durations, followed by sample rehydrogenation in plasma.

Parameter	Untreated	High-Temperature Treated and Rehydrogenated			
		3 min	10 min	30 min	90 min
i_A_/mA	0.44	0.39	0.26	0.29	0.24
i_A_/i_C_/-	0.97	1.00	1.19	0.88	1.06
ΔE/V	0.35	0.22	0.50	0.55	0.20
